# New Frontiers in Child, Adolescent and Young Adult Psycho-Oncology Survivorship Care

**DOI:** 10.3390/cancers14184335

**Published:** 2022-09-06

**Authors:** Ursula M. Sansom-Daly, Jordana K. McLoone, Lauren Touyz, Christina Signorelli

**Affiliations:** 1 Behavioural Sciences Unit, Kids Cancer Centre, Sydney Children’s Hospital, Randwick, NSW 2031, Australia; 2 Discipline of Paediatrics & Child Health, School of Clinical Medicine, Randwick Clinical Campus, UNSW Medicine & Health, UNSW Sydney, Kensington, NSW 2052, Australia; 3 Sydney Youth Cancer Service, Nelune Comprehensive Cancer Centre, Prince of Wales Hospital, Randwick, NSW 2031, Australia

The landscape of cancer survivorship has changed considerably from Fitzhugh Mullan’s conceptualization of the three sequential phases or ‘seasons of survival’ that an individual might expect to pass through, from the acute (cancer diagnosis and treatment), extended (the period following treatment), and permanent (survivorship, aligned with cure) survivorship phases [1]. In the 1980s, Mullan’s advocacy for a move away from cancer ‘victimhood’ towards ‘survivorship’ through the National Coalition for Cancer Survivorship marked a critical societal shift in how cancer was viewed and experienced. This original definition was broad in the sense of enabling anyone living with cancer to identify as a ‘cancer survivor’—from the moment of diagnosis up until and including the end of life.

However, these seasons of survival offer a picture of survivorship that is arguably more black-and-white than the lived experiences of many young people and their families being diagnosed with, and treated for, cancer in 2022 [2]. Advancements in the field of oncology (such as precision medicine) and psychosocial care, including examples published in this Special Issue, necessitate careful reflection on the potential changes that are needed to account for the increasing diversity of what it means to be a cancer survivor. During this exciting time of change and advancement, we need to reflect optimistically on opportunities for psycho-oncology research to evolve—both in its focus and its methodologies—to meet the new and emerging challenges in child, adolescent and young adult (CAYA) cancer care. This article discusses several of the ways in which medical advances have changed cancer treatment and patient outcomes, and how these changes may impact psycho-oncology care and research. Through the lens of this new era, we offer observations about the ways in which psycho-oncology research and practice needs to continue to evolve to ensure the best outcomes for young people, families, and the healthcare professionals and systems involved in caring for them.

## 1. The Changing Landscape of Psycho-Oncology Care in Survivorship: Precision Medicine

The use of precision medicine to treat cancer is arguably the most exciting development in CAYA oncology in recent decades. Precision approaches to CAYA cancer treatment involve using novel diagnostic processes to first identify the presence of specific pathogenic molecular aberrations and then match a tailored medical treatment strategy to best target these [3,4]. This is a paradigm shift from traditional models of treatment, which typically use a standard protocol according to each cancer type. Precision oncology has led to new hope for some groups of young people living with high-risk cancers that a ‘cure’ is possible [5,6].

The ongoing expansion of access to new precision medicine trials means that for many young people, their experience of cancer may be experienced as less of an acute illness and rather as more of a chronic illness, potentially remitting/relapsing in nature, with the real possibility of living with metastatic disease or receiving treatment long term. Existing at the edge of this new frontier in precision medicine means that for many patients and families, the opportunity for short-term remission(s) may nevertheless pave the way to a cancer that ultimately proves incurable and fatal. The ‘grey zones’ that exist in the liminal space between active/progressive disease and ‘cure’ have expanded in manifold directions [7]. Traditional survivorship programs may not yet cater appropriately to the needs of young people who may be striving to engage fully in developmentally normal lives whilst living with (potentially) incurable disease [8,9]. What it means to be a ‘cancer survivor’ has changed, with the nature and direction of these changes for each individual young person likely to be as diverse as the genetic anomalies we are now capable of diagnosing and targeting.

## 2. Achieving More Personalized Psychosocial Care in a New Era of Medicine

In clinical and research practice, a more nuanced approach to how we first define, and then study, ‘cancer survivors’ is likely to become necessary. The use of broad definitions such as having completed treatment with ‘curative intent’ may become unsuitable [10,11], while increasingly patients are ‘surviving-with’ cancer [7]. Similarly, it may become increasingly meaningless to group survivors in traditional ways when, for example, designing psychosocial interventions to meet their needs, or when evaluating interventions, given the diversity of cancer-treatment-related risk factors that are likely to impact the psychological terrain of a young person’s cancer experience. Survivors diagnosed with the same overarching cancer type may receive quite different treatments should one or both receive personalized medicine for particular targetable genetic mutations, with different short- versus long-term prognostic outlooks. The diversity of survivorship experiences and outlooks will create new demands for practising psycho-oncologists to deliver tailored interventions that are experienced as supportive and safe—particularly in the peer-group setting where two young people or family members’ experiences may differ markedly [12]. This may be particularly challenging within a field that is also trying to increase access to economically efficient, scalable, and evidence-based psychosocial interventions, which are often standardized and manualized in nature [13].

## 3. Understanding Mechanisms of Risk and Protective Factors

The challenge to continue to develop cost-efficient, scalable interventions may lead the field to develop new psycho-oncology intervention models that support greater flexibility, such as selecting relevant components of an intervention to align with an individual’s unique set of risk factors. Traditional goals of simply reducing distress at a designated time-point may be replaced by interventions designed to enhance young people’s underlying skills for adaptive cognitive, emotional, and behavioural processes, to ensure critical age-appropriate, positive real-world functioning that is generalizable across their cancer journey [14,15]. In recognition that increasingly, distress may develop anywhere along the continuum of survival, establishing eligibility or inclusion criteria based on predefined risk factors (e.g., recently off treatment) may not be easy and designing interventions that instead offer life-long education and skills-based training may be warranted.

## 4. Acknowledging Diversity in Survivorship

For researchers, addressing survivors’ diverse experiences and needs increases the already challenging aspect of recruiting sufficiently homogenous, large sample sizes for psychosocial studies among relatively rare CAYA cancers. The need for more nuanced psychosocial care means that developing and evaluating scalable interventions will become more challenging and will impact the translation and strength of evidence available to inform clinical care. Greater collaborations between researchers, clinicians and survivors in iterative processes of co-design are needed now more than ever. The next generation of psycho-oncology interventions will likely draw upon valuable patient and consumer partnerships to tailor evidence-based interventions and direct interventions to those most at need and, importantly, define when psychosocial support is needed [16].

## 5. All Things Digital: Telehealth Opportunities and Challenges

The burgeoning availability and rapid uptake of new digital technologies, fuelled by the global coronavirus (COVID-19) pandemic [17], may provide some new solutions to this challenge of meaningfully clustering survivors and families together. Traditionally, psychological support interventions often adopt a transdiagnostic approach—that is, including all-comers regardless of their specific cancer diagnosis—to ensure a critical mass of participants [13]. This is especially likely in AYA cancer survivorship, where there is a lack of critical mass of AYA survivors treated across diverse paediatric and adult cancer sites. When group-based models of support are used that harness the benefits of peer connection to provide support and improve people’s care experiences this can mean young people are exposed to the cancer-related circumstances and experiences of individuals with considerably different diagnoses, prognoses, and treatments [11]. While young people and their families often demonstrate capacity to connect emotionally despite their different cancer experiences [11,18,19], in this new era of medicine, it is increasingly likely that the experiences of two adolescents with the same diagnosis may diverge in important ways. It is worth considering the ways in which digital-delivery mechanisms might facilitate diagnosis-specific psychosocial interventions to be developed and delivered to small and specific sub-groups of patients, survivors and their family members [20]. The hope is that through greater collaborations with technology companies, greater efficiencies may be built into the clinical workforce and more artificial intelligences can be built into program design. In this way, standardized programs may still be individualized if rules and logic are able to construct a psychological package that matches to the individual’s personal profile [21]. This is a leap from traditional manualized interventions that are offered as a one size fits all model.

## 6. Upskilling Healthcare Professionals to Meet the Demands of the Changing Landscape

The new paradigm of psycho-oncology care that we argue needs to accompany advances in treatment requires new knowledge and skills and imposes new emotional demands on the multidisciplinary healthcare professionals involved in caring for CAYAs. It would be short-sighted to expect this care to continue to be truly personalized, child- and family-centred, and of a gold standard without considering how our CAYA oncology workforce may need to be better equipped and upskilled to adeptly navigate this new terrain. The number of patients who are surviving for longer and living with cancer in more chronic ways will continue to test our ability to cater for their psychosocial needs across time and as they disperse geographically.

The limits of resources available to deliver high-quality psycho-oncological care in survivorship in cost-constrained hospital settings will continue to warrant innovative solutions in the field to enable evidence-based interventions to be implemented at scale; this may include training nursing staff to facilitate interventions to improve psychological outcomes and healthcare engagement among long-term cancer survivors [22], or by training community-based cancer support counsellors to deliver intensive, group-based psychological programs via telehealth for survivors [11] or their family members [19]. However, the COVID-19 pandemic highlighted that despite its great potential, there are also limits to what can be achieved using digital technologies and distance-delivered interventions [23]. To realize the potential of digitally delivered psycho-oncology interventions, greater care will need to be paid to supporting health professionals to manage the more nuanced ‘human factors’ involved in delivering high-quality psycho-oncology care with all the new challenges and constraints associated with digital delivery [23].

## 7. Supporting Healthcare Providers: The Risk of Burnout

There has also been a qualitative shift in the experience of cancer survivorship, and this also poses important challenges to the resilience of the whole workforce. The prognostic uncertainty and frequent challenges and heartbreaks associated with precision medicine, for example, involves the potential for considerable emotional burden among clinicians and scientists working on precision medicine trials [24,25]. Burnout among CAYA oncology professionals is a threat to the workforce—and to the delivery of quality cancer care—that is as yet understudied [25]. Yet, the growing number of patients who receive a guarded or uncertain prognosis and move into a chronic ‘meta-vivorship’ are also going to need cancer care professionals to be emotionally prepared, skilled and confident to support patients around their realistic fears of cancer progressing, ongoing financial toxicity, and to move in and out of supportive and palliative care and end-of-life relevant conversation topics at different points of the cancer care continuum [26]. Although CAYA oncology health professionals currently report feeling a lack of confidence in these types of communication, these skills will be needed by the entire multidisciplinary healthcare team in order for age-appropriate standards of care to be maintained [26,27,28].

## 8. Extending Support to Community Healthcare Providers

As the survivorship cohort grows, increasing pressure is being applied to transition low–medium-risk survivors to a shared care model, or with complete discharge to general practitioner (GP)-led care. While a traditional challenge, with considerable literature suggesting GPs are not confident to manage the unique care needs of survivors of rare disease (as each of the childhood cancers are), this challenge is exacerbated by the increasing divergence in survivors’ risk of developing late effects, as new therapies, immunotherapies and precision medicine protocols emerge [29]. Without clear guidance, it is unrealistic for GPs to remain knowledgeable across this plethora of possible outcomes and treat patients in line with emerging and changing medical, screening, health behaviour, and mental health guidelines. New efficiencies in artificial intelligence (AI) may be exploited to find innovative ways to connect GPs with ‘live’ survivorship guidelines and care plans, rather than traditional, static documents that rapidly become outdated. Similarly, these new AI technologies may efficiently retain survivors in tertiary-level care for low-cost, routine comprehensive health assessments (completed by the survivor online), with escalation to the GP accompanied by specialist recommendations, should the health assessment trigger an auto-alert [22].

## 9. Prolonged Periods of ‘Surviving-with’: The Long-Term Impact

With more patients experiencing a longer, more chronic cancer course, psycho-oncology needs to adjust alongside medicine to examine mental health outcomes beyond the five-year survival mark, as patients are more often ‘surviving-with’ cancer [7]. Whilst we have observed a steady decline in deaths overall among long-term survivors, the risk of death beyond five years has increased for some survivors in more recent decades; for example, an increasing number of young patients with central nervous system tumours are surviving, yet also living with more long-term, life-threatening cancer-related late effects [30,31]. In clinical practice, this is already changing the prevalence and types of psychological distress that are seen. The new concept of ‘meta-vivorship’ attempts to capture the existence of living and surviving with a metastatic, likely ultimately incurable, cancer [9]. Clinically, fear of cancer recurrence will need to incorporate fear of cancer progression for those individuals [32]—and their families [33]—where the cancer was never completely gone but is simply being held at bay.

In addition, we must find new ways to ensure childhood cancer survivors who have transitioned to adult, non-cancer care (e.g., an adult neurologist after completing childhood brain cancer treatment) continue to be assessed and treated for latent cancer-related distress. Examples of this latent distress include cases of cancer-related infertility, diagnosed a decade after the completion of treatment, or the grief and bereavement of a family when a young person dies decades post-treatment due to cancer-related late effects, years before their ‘time’. As the span of morbidity and mortality extends into the future, the associated psychological issues will be critical to address with evidence and advocacy for the greater inclusion of, and funding for, a workforce with the capacity to meet the mental healthcare needs of the cohort. Research evaluating various models of care, with health economics evaluations embedded in the design, are needed to inform how we can identify optimal models of care that are sustainable as survivorship numbers grow and mortality is shifted in some cases to a later phase of the cancer patient’s journey.

## 10. Conclusions

It is an exciting time for psycho-oncology researchers and clinicians alike. Advances and resulting shifts within the field of medicine and digital technology require those of us working within the field of psycho-oncology to rise to the challenges of our own new frontiers. Psycho-oncology care requires innovation to keep pace with the shifting landscape and emerging needs of patients and survivors. Yet, in realizing the vision of psycho-oncology research and practice that meets the challenges of survivorship remodelled by these innovations, there remain numerous barriers that we need to address. Our survivorship care models and approaches require as much personalization as the medical treatments patients now receive [34]. Realizing improved patient outcomes has never been more important for patients who discover their cancer so young and who have the potential to look forward to a long and promising life—while surviving with, and beyond, their cancer.
